# Small Nuclear RNAs (U1, U2, U5) in Tumor-Educated Platelets Are Downregulated and Act as Promising Biomarkers in Lung Cancer

**DOI:** 10.3389/fonc.2020.01627

**Published:** 2020-08-12

**Authors:** Xiaohan Dong, Shanshan Ding, Miao Yu, Limin Niu, Linlin Xue, Yajing Zhao, Li Xie, Xingguo Song, Xianrang Song

**Affiliations:** ^1^Department of Clinical Laboratory, Shandong Cancer Hospital and Institute, Shandong First Medical University & Shandong Academy of Medical Sciences, Jinan, China; ^2^Department of Clinical Laboratory, Shandong Provincial Third Hospital, Cheeloo College of Medicine, Shandong University, Jinan, China; ^3^Shandong Provincial Key Laboratory of Radiation Oncology, Shandong Cancer Hospital and Institute, Shandong First Medical University & Shandong Academy of Medical Sciences, Jinan, China

**Keywords:** small nuclear RNA (snRNA U1, U2, U5), tumor educated platelet, lung cancer, biomarkers, alternative splicing

## Abstract

**Background:**

Small nuclear RNA (snRNA) levels are extremely variable across a wide range of biological conditions. SnRNAs could potentially regulate alternative splicing to drive genetic, dysplastic and neoplastic disease, which might be the main reason for mRNA profile alteration in tumor educated platelets (TEPs).

**Methods:**

Platelets were isolated from the plasma of lung cancer patients and healthy donors by low-speed centrifugation and subjected to RNA isolation. SnRNA U1, U2, U5 levels were detected by quantitative real-time polymerase chain reaction (qRT-PCR). Exosomes were isolated by ultracentrifugation and identified by qNano.

**Results:**

TEP U1, U2, U5 levels were significantly decreased in patients with lung cancer as well as with early stage patients, their downregulation was correlated with lung cancer progression, possessing favorable diagnostic efficiency. More importantly, TEP U1, U2 and U5 levels were closely correlated between paired exosomes and TEP from treated patients but not from untreated ones, and U1, U5 but not U2 in platelets were elevated by apo-exosomes.

**Conclusion:**

Tumor educated platelet small nuclear RNAs are downregulated and act as promising biomarkers in lung cancer.

## Background

Lung cancer is the most common cause of cancer-related death worldwide, with an average 5-year survival rate of 15% (1). Unfortunately, symptoms of lung cancer are obscured until the patients reach the end stage of the disease, and approximately two−thirds of patients present with metastatic tumors at the time of diagnosis (2), reflecting the urgent need to develop the reliable biomarkers for the early diagnosis of lung cancer and the monitoring of its spatial and temporal progression.

Platelets, originating as anucleate cytoplasts from megakaryocytes (3), play crucial roles not only in hemostasis, but also in systemic and local responses to the presence of cancer (4), thereby sequestering tumor-specified biomolecules including RNA transcripts and proteins, as well as altering their spliced RNA profiles, called “tumor educated platelets (TEPs)” (5). Recently, TEPs emerged as non-invasive biomarker source for cancer detection and progression monitoring including colorectal carcinoma (CRC) (6), glioblastoma (7), non–small cell lung cancer (NSCLC) (8), prostate cancer (9) and etc., due to their significantly altered RNA profiles. Moreover, RNA profiles in TEPs enabled to discriminate patients with localized and metastasized cancer from healthy controls, even precisely pinpoint the primary origin of pan-cancer, as well as predict the oncogenic status including *MET* or *HER2* positivity and the existence of *KRAS*, *EGFR*, or *PIK3CA* mutations (10). Due to the lifespan of 7–10 days and the structure of platelet membrane, tumor-specified biosources and biomolecules are enriched in the TEPs and protected from circulating RNAs, and thus TEP RNA analysis is capable to reflect tumor bioactivity up-to-date, intensively, and dynamically.

Platelet lacks a nucleus, thus no genomic DNA is available for transcription of new RNA molecules, but endogenous pre-mRNAs can be spliced into mature mRNAs (11). Specific splice events in TEPs may be induced by external stimuli such as activation of platelet surface receptors, or in response to signals released by cancer cells and the tumor microenvironment, endowing TEPs with a highly dynamic mRNA repertoire with potential applicability to cancer diagnostics (12). It has been reported mRNA profiles were significantly altered in TEPs of lung cancer, with specific spliced mRNA signature (8, 13). Spliceosome is a complex of small ribonucleoproteins (snRNPs) composed of splicing proteins and snRNAs including U1, U2, U4, U5, and U6, all of which are present in platelet (14). SnRNAs of the spliceosome are not merely the basal factors as has long been assumed, ubiquitously expressed in all cells since they are required for splicing, whereas snRNA levels are extremely variable across a wide range of biological conditions, even across all samples taken from breast ductal carcinoma (15). Endogenous variation in snRNAs could potentially enable these RNAs to regulate alternative splicing to drive genetic, dysplastic and neoplastic disease (15, 16). Therefore, we supposed snRNA levels in TEPs of lung cancer were varied from those in healthy individuals, which might the main reason for alteration of TEP mRNA profiles, exerting the potential as the biomarker for lung cancer diagnostics.

In the present study, we aimed to validate whether TEP snRNAs served as the potential biomarkers for lung cancer. We found that TEP U1, U2, U5 were significantly downregulated in lung cancer patients compared with those in healthy controls, possessing with the favorable diagnostic efficiency. Moreover, we observed that relative snRNAs expression was elevated after anticancer therapy, which seemed to attribute to apo-exosomes (apoptotic exosomes)-dependent horizontal transmission, suggesting that aberrant expression of TEP U1, U2, U5 act as novel biomarkers for lung cancer.

## Materials and Methods

### Patients and Healthy Donors

A total of 382 lung cancer patients without any anticancer treatment and 23 patients with initial treatment from Shandong Cancer Hospital and Institute, were enrolled between January 2019 to November 2019. Cancer diagnosis was determined through a histological examination of tumor specimens, and the tumor stage was determined according to the 8th edition of the lung cancer TNM staging standards formulated by IASLC; 156 healthy volunteers from the above hospital and 205 from Shandong Provincial Third Hospital were excluded from any malignant tumor after examination and enrolled in this study. All participants gave their informed consents for specimen collection and clinical information collection.

### Cell Line

The human NSCLC-derived cell line A549 was obtained from China Center for Type Culture Collection (Wuhan, China), and cultured in Dulbecco’s modified Eagle’s medium (DMEM, Gibco, Invitrogen, Carlsbad, CA, United States) supplemented with 10% fetal bovine serum (FBS, Gibco) and 100 U ml^–1^ penicillin and streptomycin (Gibco) at 37°C in a humidified 5% CO_2_ incubator.

### Platelet Isolation

Platelet isolation was performed as described previously (17). In brief, peripheral blood was collected in EDTAK2-coated purple-cap Vacutainer tubes (BD, Franklin Lakes, NJ, United States), and centrifuged at 120 × *g* for 10 min twice to separated platelet-rich plasma (PRP) from nucleated blood cells, followed by another 360 × *g* centrifugation for 20 min to pellet platelets at room temperature.

### RNA Isolation and qPCR

Total RNA was extracted with the TRIzol reagent and reverse−transcribed into complementary DNA (cDNA) using the Takara PrimeScript RT reagent Kit (Takara Bio, Kusatsu, Japan) in 20 μl reaction according to the manufacturer’s instructions. The qRT-PCR was performed using UltraSYBR Mixture (CWBio, Beijing, China) in a volume of 25 μl (12.5 μl of 2 × UltraSYBR Mixture, 0.5 μl of forward premier, 0.5 μl of reverse premier, 1 μl cDNA and 10.5 μl water). The reactions were performed with LightCycler 480 qPCR system (Roche Diagnostics, Germany). 18S acted as control as described previously (18). The relative expression of snRNAs was evaluated by the comparative cycle threshold (ΔCt) method: (ΔCt = Ct^snRNA^ – Ct^18S^) as described previously (19). The qPCR primers are listed in Table 1.

**TABLE 1:** Sequence information of the primers for qRT-PCR.

snRNA	Forward primer	Reverse primer
U1	CAGGGGAGATACCATGATCACGAAG	CGCAGTCCCCCACTACCACAAAT
U2	CCTTTTGGCTAAGATCAAGTGTAGTATCTGTT	AGCAAGCTCCTATTCCATCTCCCTG
U5	TACTCTGGTTTCTCTTCAGATCGCATAA	CTCAAAAAATTGGGTTAAGACTCAGA
18s	GGCCCTGTAATTGGAATGAGTC	CCAAGATCCAACTACGAGCTT

### Exosomes Isolation

Exosomes isolation was performed as described previously (20). Briefly, the platelet-free plasma was centrifuged at 12,000 *g* for 30 min at 4°C to exclude the cell debris, then subjected to a 0.22 μm pore filter (Millipore), followed by ultracentrifugation at 100,000 × *g* for 120 min at 4°C to harvest exosomes (Beckman Coulter, Brea, CA, United States). Isolated exosomes were resuspended with cold phosphate-buffered saline (PBS) and identified using qNano (Izon Science Ltd., Christchurch, New Zealand).

### Coincubation of Platelets With Exosomes

A549 cells were seeded in 100 mm dishes in exosome-depleted DMEM with 10% exosome-depleted FBS, treated with or without 50 μM cisplatin (Sigma-Aldrich, Shanghai, China) for 24 h. Then total 50 ml contained media were collected for exosome isolation. The amount of exosomes was measured by the BCA Protein Assay kit (Thermo Scientific, CA, United States), 100 μg exosomes were collected for qPCR or for coincubation with platelets.

Platelets were isolated from healthy volunteers and transferred to a 15 ml centrifugal tube and counted under a microscopy after washing by 0.8% EDTA-PBS. Then, 1 × 10^9^ platelets were suspended with 1 ml exosome- depleted medium and cultured with above 100 μg exosomes in EDTAK2-coated purple-cap Vacutainer tubes on a Platelet Oscillometer (L-Y70, Suzhou, China) for 4 h. After washing twice by 0.8% EDTA-PBS, platelets were collected and subjected to qPCR.

### Statistical Analysis

The data analyses were performed with GraphPad Prism version 6.0 (GraphPad Software, San Diego, CA, United States) and SPSS 22.0 software (IBM, Ehningen, Germany). The Kolmogorov–Smirnov test was carried out to check the normality of the distribution. If the data followed normal analysis, unpaired *t*-test would be used; if not, Mann-Whitney test would be used. Chi-square test was used for analyzing categorical variables. Multi-group analysis was tested by Kruskal-Wallis test. In paired data, the normally distributed numeric variables were evaluated by paired *t*-test, whereas non-normally distributed variables were analyzed by Wilcoxon rank-test. Receiving operating characteristic (ROC) curve was used to evaluate diagnostic efficiency. For correlation, if the data followed normal analysis, Pearson correlation test would be used; if not, Spearman correlation test would be used. All the values were represented as mean ± interquartile range and *p* values <0.05 were defined as statistically significance, and all tests were set as double-tailed.

## Results

### TEP U1, U2 and U5 Were Downregulated in Lung Cancer

To screen out the differential snRNAs expression levels in lung cancer, 10 snRNAs expression including U1, U2, U4, U5, U6, U7, U8, U12, U4ATAC and U6ATAC was detected in TEPs from 48 healthy donors and 48 lung cancer patients (data not shown). U1, U2, U5 were identified due to their remarkable differences and then subjected to a larger scale validation cohort including 361 healthy donors and 382 lung cancer patients naive to any anticancer treatment.

**FIGURE 1:**
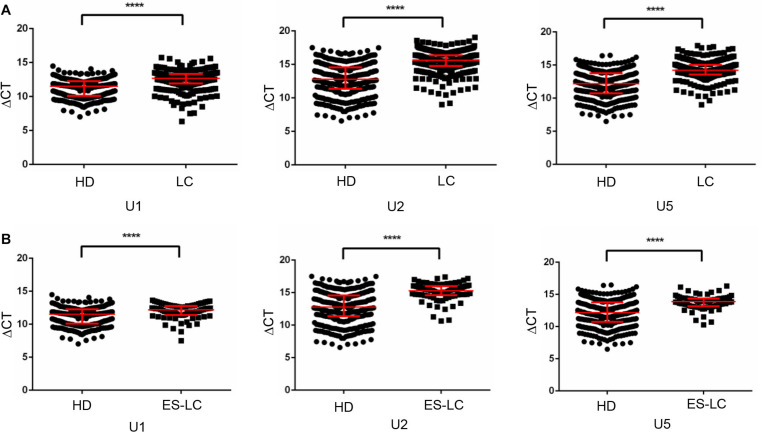
TEP U1, U2 and U5 were downregulated in lung cancer. **(A)** TEP U1, U2, U5 were down-regulated in lung cancer patients (*n* = 382) compared with healthy controls (*n* = 361); **(B)** TEP U1, U2, U5 were down-regulated in early stage lung cancer patients (*n* = 80) compared with healthy controls (*n* = 361). HD, healthy donors; LC, lung cancer patients; ES-LC, early stage lung cancer patients. ****p < 0.0001.

First, Mann-Whitney test was performed to analyze the differential expression between lung cancer patients (*n* = 382) and healthy controls (*n* = 361). TEP U1, U2, U5 were significantly decreased in lung cancer patients compared with those in healthy controls (*p* < 0.0001, *p* < 0.0001 and *p* < 0.0001, respectively) (Figure 1A), indicting the variable expression of TEP snRNAs in lung cancer. The correlation between U1, U2, U5 expression and clinicopathological characteristics was also evaluated. As shown in Table 2, U1 and U5 were obviously associated with gender and smoking and T stage, whereas U1 was associated with drinking, U5 was related to histology type and tumor size; Moreover, all these three snRNAs were closely relevant to TNM stage and lymph node metastasis, both U1 and U2 were significantly associated with distant metastasis.

**TABLE 2:** Correlation between TEP U1, U2, U5 expression and clinicopathologic characteristics of lung cancer patients.

Characteristics		Cases No. (%)	U1			U2			U5		
			Low	High	*p*	Low	High	*p*	Low	High	*p*
Gender	Male	252 (66.0)	111	141	**0.001**	120	132	0.195	116	136	**0.031**
	Female	130 (34.0)	80	50		71	59		75	55	
Age (y)	≥62	199 (52.1)	93	106	0.183	97	102	0.609	97	102	0.609
	<62	183 (47.9)	98	85		94	89		94	89	
Smoking	Yes	187 (49.0)	79	108	**0.003**	84	103	0.052	83	104	**0.032**
	No	195 (51.0)	112	83		107	88		108	87	
Drinking	Yes	126 (33.0)	47	79	**<0.0001**	58	68	0.276	61	65	0.663
	No	256 (67.0)	144	112		133	123		130	126	
Histology	NSCLC										
	AC	222 (58.1)	120	102	0.172	114	108	0.693	127	95	**0.002**
	SCC	88 (23.0)	40	48		43	45		33	55	
	Others	12 (3.1)	6	6		7	5		6	6	
	SCLC	51 (13.4)	24	27		25	26		22	29	
	Unknown	9 (2.4)									
Tumor size	≥8.788 cm^3^	145 (38.0)	69	76	0.124	70	75	0.287	64	81	**0.018**
	<8.788 cm^3^	143 (37.4)	81	62		78	65		83	60	
	Unknown	94 (24.6)									
T stage	Tis	11 (2.9)	9	2	**0.03**	8	3	0.264	10	1	**0.007**
	T1	91 (23.8)	51	40		52	39		55	36	
	T2	122 (31.9)	62	60		58	64		57	65	
	T3	44 (11.5)	24	20		21	23		21	23	
	T4	88 (23)	34	54		40	48		37	51	
	Unknown	26 (6.9)									
TNM stage	Stage 0	11 (2.9)	9	2	**0.001**	8	3	**0.021**	10	1	**0.001**
	Stage I	64 (16.8)	44	20		41	23		43	21	
	Stage II	24 (6.3)	14	10		14	10		12	12	
	Stage III	87 (22.8)	42	45		41	46		37	50	
	Stage IV	187 (49.0)	79	108		81	106		85	102	
	Unknown	9 (2.3)									
LN metastasis	Yes	253 (66.2)	107	146	**<0.0001**	116	137	**0.012**	113	140	**0.001**
	No	106 (27.7)	72	34		64	42		67	39	
	Unknown	23 (6)									
Distant metastasis	Yes	187 (49.0)	79	108	**0.002**	81	106	**0.013**	85	102	0.079
	No	187 (49.0)	109	78		105	82		102	85	
	Unknown	8 (2)									

*Chi-square test was used. Bold value, *p*< 0.05; NSCLC, non-small cell lung cancer; AC, adenocarcinoma; SCC, squamous cell carcinoma; SCLC, small cell lung cancer.

Then, we analyzed the differential expression of TEP U1, U2, U5 in 80 early stage lung cancer patients (Tis stage = 11, I stage = 64, IIA stage = 5) as well as in 361 healthy donors. Consistently, TEP U1, U2, U5 were also dramatically decreased in early stage lung cancer patients (*p* < 0.0001, *p* < 0.0001 and *p* < 0.0001, respectively) compared with those in healthy subjects (Figure 1B). Taken together, these data imply that TEP U1, U2, U5 might be involved in carcinogenesis and metastasis of lung cancer.

### TEP U1, U2, U5 Downregulation Correlated With Lung Cancer Progression

**FIGURE 2:**
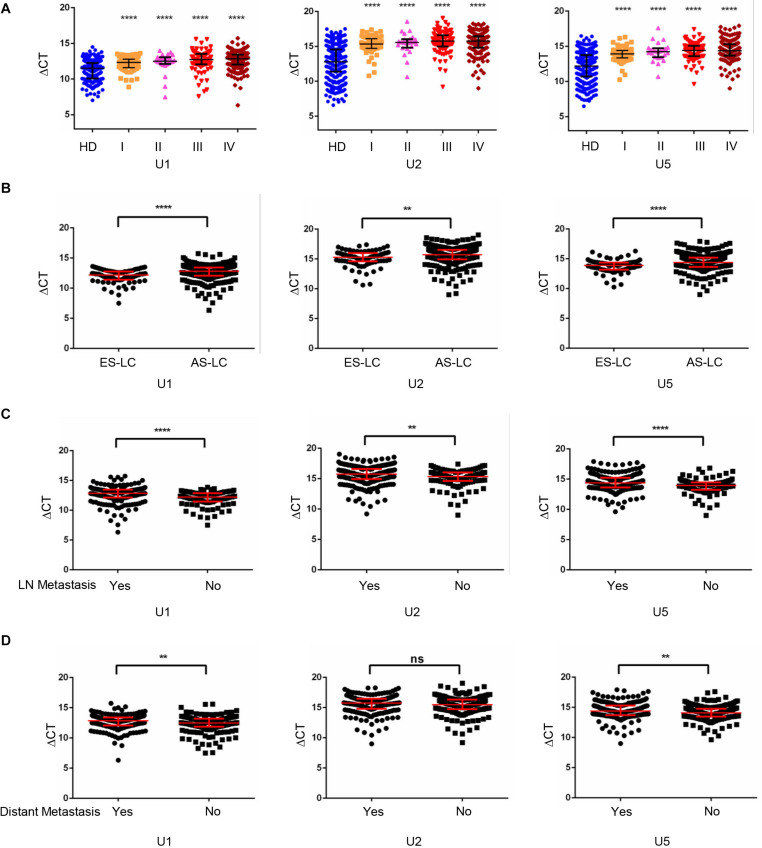
TEP U1, U2, U5 downregulation correlated with lung cancer progression. **(A)** TEP U1, U2, U5 were down-regulated in in various TNM stage; **(B)** TEP U1, U2, U5 were down-regulated in advanced-stage lung cancer patients (*n* = 274) compared with early-stage patients (*n* = 80); **(C,D)** TEP U1, U2, U5 expression was lower in lymph node metastasis **(C)** and distant metastasis **(D)** group than in control group. AS-LC, advanced-stage lung cancer patients; **p < 0.005; ****p < 0.0001; ns, no significance.

Furthermore, we analyzed the expression levels of TEP U1, U2, U5 among different stages of lung cancer patients and healthy donors. As shown in Figure 2A, when compared with the healthy donors, the TEP U1, U2, U5 expression was significantly down-regulated in various TNM stage. Next, we retrospectively analyzed the differential expression of TEP U1, U2, U5 in 80 early stage lung cancer patients (Tis stage = 11, I stage = 64, IIA stage = 5) and 293 advanced-stage lung cancer patients (IIB stage = 19, III stage = 87, IV stage = 187). As shown in Figure 2B, their expression was also significantly downregulated in advanced-stage lung cancer patients (*p* < 0.0001, *p* = 0.0013 and *p* < 0.0001, respectively) compared with those early stage lung cancer patients. Similarly, TEP U1, U2, U5 levels were much lower in lymph node metastasis while U1, U5 levels were lower in distant metastasis group than those in control group, respectively (Figures 2C,D). Collectively, our data support TEP U1, U2, U5 downregulation correlates with lung cancer progression.

### TEP U1, U2 and U5 as Biomarkers for Lung Cancer Diagnosis

**FIGURE 3:**
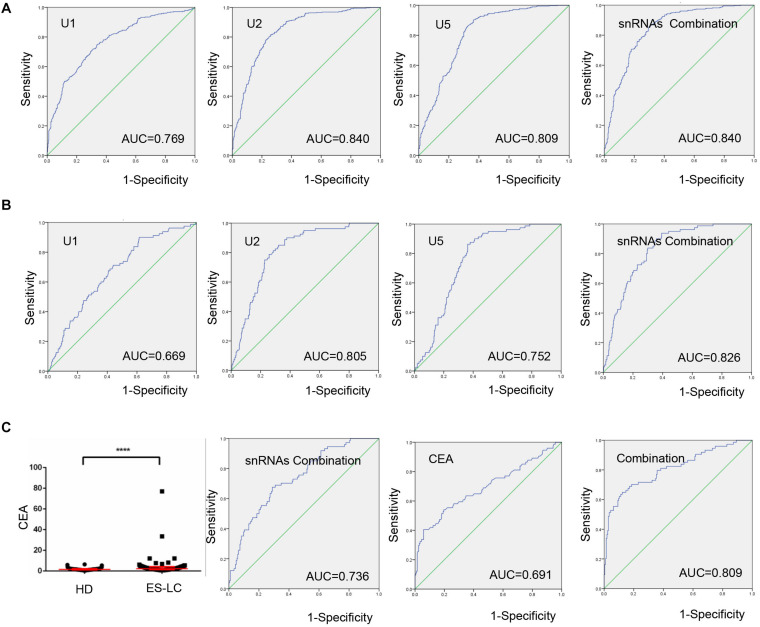
TEP U1, U2 and U5 as biomarkers for lung cancer diagnosis. **(A)** The ROC curve analysis of U1, U2, U5 and their combination for lung cancer; **(B)** The ROC curve analysis of U1, U2, U5 and their combination for early stage lung cancer; **(C)** The levels of CEA were increased in early stage lung cancer patients (*n* = 74) compared with healthy controls (*n* = 204). The ROC curve analysis of snRNAs combination, CEA and the combination of snRNAs and CEA (AUC = 0.809) for early stage lung cancer. AUC, area under curve; ****P < 0.0001.

To explore the potentiality of TEP U1, U2, U5 as circulating diagnostic markers for lung cancer, ROC curves were employed. As shown in Figure 3A, the areas under the curve (AUCs) of TEP U1, U2 and U5 were 0.769 with 74.6% sensitivity and 66.5% specificity, 0.840 with 81.4% sensitivity and 74.2% specificity, and 0.809 with 90.1% sensitivity and 63.7% specificity, respectively. Moreover, the diagnostic efficiency of their combination was also calculated, possessing AUC of 0.840 with a relative sensitivity of 85.9% and a relative specificity of 70.1%, indicating TEP U1, U2 and U5 potentially act as the non-invasive circulating biomarkers for lung cancer.

Subsequently, when comparing the patients with early stage lung cancer to healthy controls, ROC curves demonstrated favorable diagnostic efficiencies of TEP U1, U2, U5, possessing AUCs of 0.669 with 90.0% sensitivity and 38.5% specificity, 0.805 with 78.8% sensitivity and 74.2% specificity, and 0.752 with 86.3% sensitivity and 63.7% specificity, respectively. Moreover, the diagnostic performance for their combination demonstrated the AUC of 0.826 with a relative sensitivity of 93.8% and a relative specificity of 60.7% (Figure 3B).

Carcinoembryonic antigen (CEA) is a widely used blood biomarker for lung cancer diagnosis since 1980 (21), but possesses the poor clinic diagnostic efficiency at the early stage of cancer development (22). We detected the CEA levels in 74 early stage lung cancer patients and 204 healthy subjects, which were obviously higher in early stage lung cancer patients (*p* < 0.0001) than those in healthy controls (Figure 3C). Importantly, combination of TEP U1, U2, U5 significantly facilitated the diagnostic efficiency for lung cancer of CEA, the AUC of CEA was elevated from 0.691 to 0.809 with a sensitivity of 64.9% and specificity of 87.7%. In summary, TEP U1, U2, U5 potentially act as biomarkers for lung cancer diagnosis.

### TEP U1, U2, U5 as Biomarkers for Lung Cancer Progression Monitoring

**FIGURE 4:**
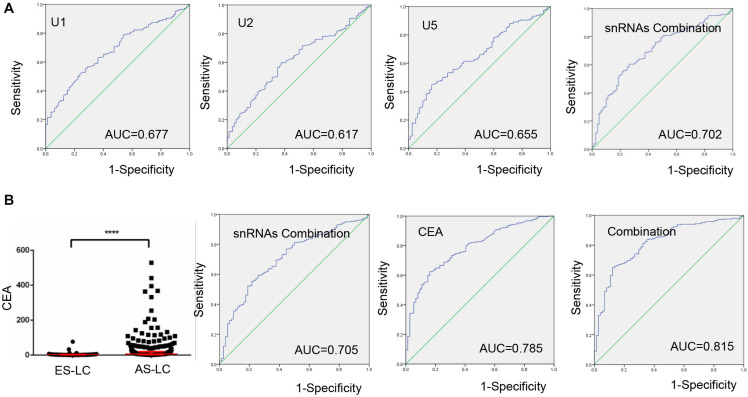
TEP U1, U2, U5 as biomarkers for lung cancer progression monitoring. **(A)** The ROC curve analysis of U1, U2, U5 and their combination for lung cancer progression; **(B)** The ROC curve analysis of CEA for lung cancer progression combined with U1, U2, U5; ****P < 0.0001.

Next, we investigated whether TEP U1, U2, U5 could serve as biomarkers to monitor lung cancer progression, and ROC curve was applied to analyze the diagnostic efficiency between early-stage and advanced-stage lung cancer patients. As shown in Figure 4A, AUCs of TEP U1, U2, U5 and their combination were 0.677 with 51.9% sensitivity and 76.25%specificity, 0.617 with 59.7% sensitivity and 62.5% specificity, and 0.655 with 44.7% sensitivity and 83.75% specificity, as well as 0.702 with 60.4% sensitivity of and 73.75% specificity, respectively, indicating that TEP U1, U2, U5 levels might be used to monitor lung cancer progression.

We also combined TEP U1, U2, U5 with CEA to evaluate their roles in monitoring cancer progression. Consistent with previous reports, CEA level was upregulated in 282 advantaged-stage compared with that in 74 early-stage lung cancer (Figure 4B). Besides, we demonstrated that the combination of TEP U1, U2, U5 and CEA improved the diagnostic efficiency of cancer progression, the AUC was 0.815 with 64.9% sensitivity and 87.8% specificity, higher than that for CEA alone (AUC = 0.785, with 62.4% sensitivity and 83.8% specificity), indicating the potential roles of TEP U1, U2, U5 in lung cancer progression monitoring.

### Platelet-Count Dependent Expression of U1, U2 and U5 Was Elevated by Anticancer Therapy

**FIGURE 5:**
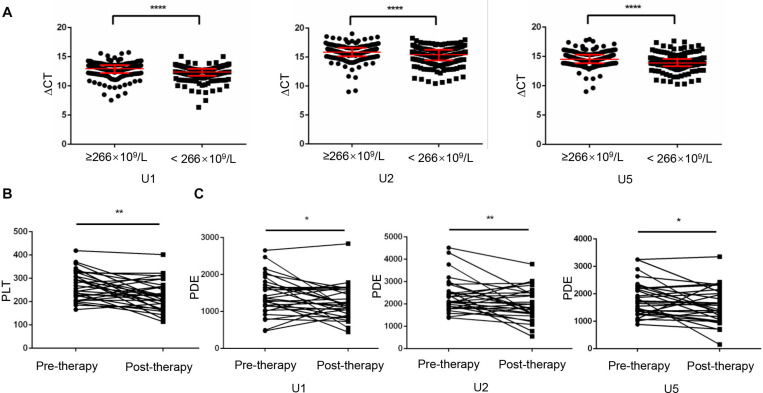
Platelet-count dependent expression of U1, U2 and U5 was elevated by anticancer therapy. **(A)** TEP U1, U2, U5 expression was down-regulated in high platelet counts patients (*n* = 192) compared with that in low platelet counts patients (*n* = 189); **(B)** The platelet count levels were significantly lower in the post-therapy patients than in paired pre-therapy patients (*n* = 30); **(C)** The PDE levels were significantly lower in the post-therapy patients than in paired pre-therapy patients; *P < 0.05; **P < 0.005; ****P < 0.0001.

Increased platelet counts could accompany various cancers including lung cancer (3), revealed as a predictor for prognosis of lung cancer (23). Therefore, in current study, we divided lung cancer patients into high (*n* = 192) and low (*n* = 189) level groups based on their median value 266 × 10^9^/L of platelet counts. As shown in Figure 5A, TEP U1, U2, U5 levels were dramatically downregulated in the patients with higher platelet counts (*p* < 0.0001, *p* < 0.0001 and *p* < 0.0001, respectively).

Given that anticancer therapy was capable to induce a decrease (*p* = 0.0016) in platelet counts (Figure 5B), snRNAs expression per platelet was elevated by anticancer therapy much more significantly, as evidence from that platelet-count dependent expression (PDE, ΔCT × platelet counts) of U1, U2 and U5 was downregulated (Figure 5C) after anticancer therapy (*p* = 0.0495, *p* = 0.0098 and *p* = 0.0276, respectively).

### TEP U1, U5 but Not U2 Expression Was Affected by Apo-Exosomes

**FIGURE 6:**
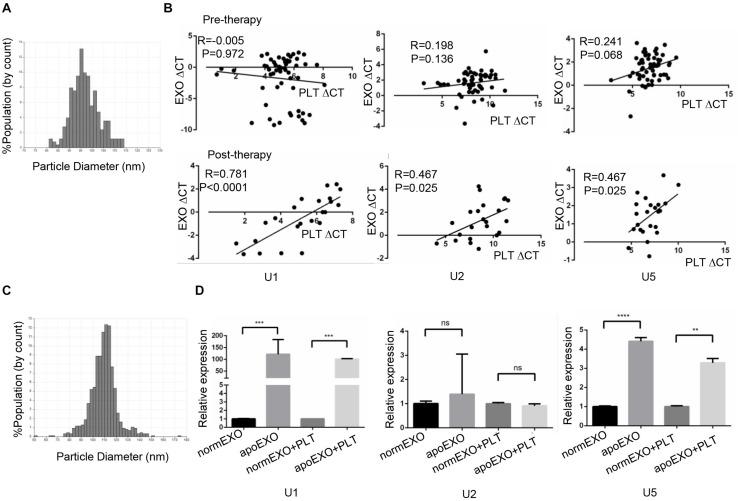
TEP U1, U5 but not U2 expression was affected by apo-exosomes. **(A)** Distribution of exosomes of 50–150 nm diameter in the samples from lung cancer patients’ plasma based on the qNano system; **(B)** The correlations between U1, U2, U5 expression and platelet counts in 58 untreated patients (upper) and 20 treated lung cancer patients (down); **(C)** Distribution of exosomes of 50–150 nm diameter in the samples from A549 cells based on the qNano system; **(D)** Exosomal U1, U5 from apoptotic A549 cells (apoEXO) were significantly increased compared to those derived from normal A549 cells. normEXO/apoEXO, exosomes derived from normal/apoptotic A549 cells. **p < 0.005; ***p < 0.001; ****p < 0.0001; ns, no significance.

Previous study had been reported chemotherapy-induced apoptosis might lead to significant release of apoptotic extracellular vesicles (apoEVs), in which snRNAs specifically were enriched (24, 25). We supposed apoEVs could be captured by TEPs, thus causing an increase of TEP snRNAs expression. Hence, we hypothesized a correlation of snRNAs level between in exosome and in TEP. To prove this hypothesis, 58 lung cancer patients before receiving any anticancer treatment (untreated) and 23 patients with initial anti-cancer therapy (treated) were enrolled, then the paired TEPs and exosomes from the same donor were collected. First, the exosomes were identified by qNano, the diameters of most exosomes concentrated on the 50–150 nm (Figure 6A). Then these three snRNAs were detected in paired TEPs and exosomes. As show in Figure 6B, all TEP U1, U2 and U5 levels were closely correlated between exosomes and TEPs in treated patients (*R* = 0.781, *p* < 0.0001; *R* = 0.467, *p* = 0.025; *R* = 0.467, *p* = 0.025, respectively), but not in untreated ones unexpectedly. These data support anti-cancer therapy induced apo-exosomes might transmit snRNAs to TEPs.

To confirm the transmission of snRNAs to platelets by apo-exosomes, A549 cells were employed and treated with or without 50 μm cisplatin for 24 h to induce apoptosis. A549 derived exosomes were isolated and identified using qNano system (Figure 6C), followed by co-culture with platelets from healthy volunteers for 4 h. As shown in Figure 6D, exosomal U1, U5 from apoptotic A549 cells (apoExo) were significantly increased (*p* = 0.0008 and *p* < 0.0001, respectively) compared to those from normal A549 cells (normEXO). In consistence, U1, U5 in platelets were also significantly upregulated (*p* = 0.0333 and *p* = 0.005, respectively) after coincubation with exosomes from apoptotic A549 cells (apoEXO + PLT) than that from normal A549 cells (normEXO + PLT). Unexpectedly, no significant differences of U2 was observed neither in normEXO nor in apoEXO, as well as neither in normEXO + PLT nor in apoEXO + PLT.

## Discussion

Despite continuous improvements of therapy strategies, the mortality rate of lung cancer patients still remains high, mainly since most patients were diagnosed at an advanced-stage (24). Thus, sensitive and specific biomarkers to identify lung cancer patients are urgently needed. Platelets, “educated” by their environment, contain a rich and dynamical repertoire of RNA varieties, including mRNAs and small non-coding RNAs, thus providing the biosources and biomolecules as diagnostic, prognostic, predictive or monitoring biomarkers. In our study, we demonstrated TEP U1, U2, U5 served as potential biomarkers for diagnosis and progression monitoring for lung cancer.

Splicing usually occurs within nuclei, yet platelets devoid of a nucleus do possess essential splicing factors (25), including all of the five snRNAs (U1, U2, U4, U5 and U6), which were detected in nucleus and cytoplasm of megakaryocytes, and in proplatelets that extend from megakaryocytes, as well as in primary circulating platelets (26) implementing signal-dependent splicing, a unique splicing mechanism upon platelet activation by tissue factor (27) or clooxygenase-2 (Cox-2) (28), providing platelets with a novel mechanism to alter their mRNA profile. The U1, approximately 165 nucleotides in length, participates in splicing by recognizing and binding to 5′ splice sites (SS) at pre-mRNA exon/intron boundaries to form the E complex (29); U2 forms part of the RNA network to bring the reactive sites into approach of pre-mRNA at the catalytic core of spliceosome; U5 snRNA interacting with the 5′ exon is thought to assist in docking the 5′ SS into catalytic core of the spliceosome (30). Our data validated TEP U1, U2, U5 as potential biomarkers for lung cancer. Firstly, TEP U1, U2, U5 expression levels were decreased significantly in patients with lung cancer or early stage lung cancer. The ROC curve analysis showed TEP U1, U2, U5 could distinguish patients from healthy donors, possessing favorable diagnostic efficiencies. Then, TEP U1, U2, U5 downregulation was correlated with lung cancer progression. They were significantly downregulated in advanced-stage, distant metastasis or lymph node metastasis lung cancer patients compared with those in control groups, respectively. The ROC curve analysis demonstrated TEP U1, U2, U5 could distinguish advanced-stage patients from early-stage ones with favorable diagnostic efficiencies.

The snRNA U4 and U6 also play crucial roles in splicing behavior of nucleated cell. During splicing, U4 and U6 interact with one another by RNA-RNA base pairs, at the time U4/U6 and U5 snRNPs are recruited as a preassembled U4/U6-U5 tri-snRNP, and interact with the pre-mRNA (31). In our study, we also analyzed the differential expression of TEP U4 and U6 between healthy donors and lung cancer patients, but no significance was observed (data not shown), indicating the different roles of U4 and U6 in platelet. In addition, second minor snRNAs including U11, U12, U4ATAC and U6ATAC which represent functional counterparts of the major U1, U2, U4, and U6 (29), were also detected in our research. However, most of them expressed very low in platelets, which are inappropriate to act as the potential biomarkers.

Interestingly, snRNAs U1, U2 and U5 levels were closely correlated between paired exosomes and TEP from treated patients but not from untreated ones, this was probably because apo-exosomes were accumulated during anticancer therapy, in which splicing messages were enriched, and then captured by TEPs, thus causing increases of TEP U1 and U5 expression. Nevertheless, U2 seemed not to be assembled in platelets as well as in apo-exosomes, implying another mechanism might be involved.

Accumulating evidences have highlighted that platelets acted as active players in all steps of tumorigenesis, and coordinated with tumor cells by both local and distant crosstalk. One of the mechanisms by which platelets are educated by tumor cells is receptor-ligand interaction. For example, pdoplanin, expressed on cancer associated fibroblasts and tumor cells, is a major ligand to attract platelets by binding to C-type lectin-like receptor 2 (CLEC-2), which has been identified as a receptor for the platelet activating snake venom protein rhodocytin, then facilitates hematogenous cancer metastasis and cancer associated thrombosis (32). In current study, we proved that horizontal transmission by apo-exosomes played a crucial role in the process that tumor educated platelets. However, we can’t exclude the possibility that direct interaction of tumor-platelet induced TEP U1, U2, U5 downregulation, which need more exploration.

However, several limitations should be carefully considered in the present study. First, our results included 382 lung cancer patients, the total sample sizes were small and might lack statistically vigorous power. Long-term clinical follow-up data were also absent, which currently limit the ability to explore the prognostic values of TEP U1, U2, U5. Moreover, the mechanisms of enrichment and transmission of exosomal snRNAs were still unclear, so the detailed process that cancer educated platelets still need more investigation.

## Conclusion

In summary, our findings reveal that TEP U1, U2, U5 were significantly downregulated in lung cancer, and their downregulations were correlated with lung cancer progression, possessing favorable diagnostic efficiencies. More importantly, TEP U1, U2 and U5 levels were closely correlated between paired exosomes and TEP from treated patients but not from untreated ones, and U1, U5 but not U2 in platelets were elevated by apo-exosomes, thus providing the evidence TEP U1, U2, U5 as biomarkers for lung cancer.

## Data Availability Statement

The raw data supporting the conclusions of this article will be made available by the authors, without undue reservation.

## Ethics Statement

The studies involving human participants were reviewed and approved by Shandong Cancer Hospital Affiliated to Shandong First Medical University and Shandong Academy of Medical Sciences of Committee. The patients/participants provided their written informed consent to participate in this study.

## Author Contributions

XRS and XGS designed the experiments. XD and SD carried out the experiments. MY provided the blood samples. XD and XGS wrote the manuscript and prepared the figures. LXi, LN, LXu, and YZ contributed to analysis the experimental data. All authors reviewed the manuscript read and approved the final manuscript.

## Conflict of Interest

The authors declare that the research was conducted in the absence of any commercial or financial relationships that could be construed as a potential conflict of interest.

## Abbreviations

Δ Ct: comparative cycle threshold
ApoEVs: apoptotic extracellular vesicles
Apo-exosomes: apoptotic exosomes
AUC: area under the curve
cDNA: complementary DNA
CEA: carcinoembryonic antigen
CRC: colorectal carcinoma
DMEM: Dulbecco’s modified Eagle’s medium
FBS: fetal bovine serum
NSCLC: non-small cell lung cancer
PBS: phosphate-buffered saline
PDE: platelet-count dependent expression
PRP: platelet rich plasma
qRT-PCR: quantitative real-time polymerase chain reaction
ROC: receiving operating characteristic
SnRNA: small nuclear RNA
SnRNPs: small nuclear ribonucleoproteins
SS: splice site
TEP: tumor educated platelet.
